# Advances in Radiotherapy in the Treatment of Metastatic Spinal Lesions

**DOI:** 10.1055/s-0045-1810030

**Published:** 2025-08-18

**Authors:** Brian Guilherme Monteiro Marta Coimbra, Alice Roxo Nobre de Souza e Silva, Cauã Bhering Soares, Caio César Nogueira de Figueiredo, Manuel Salvado Macamo, Alexandre Fogaça Cristante

**Affiliations:** 1 Spine Group, Instituto do Câncer do Estado de São Paulo, Hospital das Clínicas da Faculdade de Medicina da Universidade de São Paulo, São Paulo, SP, Brazil; 2 Radiotherapy Group, Instituto do Câncer do Estado de São Paulo, Hospital das Clínicas da Faculdade de Medicina da Universidade de São Paulo, São Paulo, SP, Brazil; 3 Specialized Complementation of the Spine Group, Hospital das Clínicas da Faculdade de Medicina da Universidade de São Paulo, São Paulo, SP, Brazil; 4 Department of Orthopedics and Traumatology, Faculdade de Medicina da Universidade de São Paulo, São Paulo, SP, Brazil

**Keywords:** neoplasm metastasis, radiotherapy, spine/surgery, coluna vertebral/cirurgia, metástase neoplásica, radioterapia

## Abstract

Metastatic spinal disease has become increasingly common due to advances in systemic oncological therapies and the increase in the overall survival of cancer patients. Its treatment is palliative, but the meaning of this term has evolved and, today, consists of better and more effective condition control over longer periods. In the past, surgical and radiotherapy techniques, especially conventional external beam radiation therapy (cEBRT), were ineffective or morbid for controlling many types of tumors. Now, the emergence and advancement of stereotactic body radiation therapy (SBRT) drastically changed this scenario. Surgery for managing metastatic disease remains significant in radiotherapy-associated strategies, but its purpose and technique have undergone major updates. However, despite all technical advances, radiotherapy has toxicity and side effects that warrant consideration. In this article, we provide an updated review covering everything from history to innovations in treating spinal metastases, focusing on their benefits, indications, and clinical impact.

## Resumo

A doença metastática da coluna vertebral tem se tornado cada vez mais comum por conta do avanço das terapias sistêmicas oncológicas e do aumento na sobrevida geral dos pacientes com câncer. O tratamento dessa enfermidade é paliativo, mas a própria acepção deste termo evoluiu e hoje consiste em um melhor e mais efetivo controle da doença por períodos mais longos. Enquanto no passado as técnicas cirúrgicas e radioterápicas, principalmente a radioterapia convencional por feixe externo (cEBRT, do inglês *conventional external beam radiation therapy* ), eram ineficazes ou mórbidas para o controle de muitos tipos de tumores, hoje, com o surgimento e avanço da radiocirurgia estereotáxica corporal (SBRT, do inglês *stereotactic body radiation therapy* ), esse cenário mudou drasticamente. A cirurgia para o manejo da doença metastática ainda tem lugar em estratégias associadas com a radioterapia, mas sofreu grande atualização de propósito e técnica. Todavia, apesar de todo o avanço técnico, a radioterapia apresenta toxicidade e efeitos colaterais que precisam ser considerados. Neste artigo, fornecemos revisão atualizada que versa desde o histórico até as inovações no tratamento das metástases vertebrais, com foco em seus benefícios, indicações e impacto clínico.

## Introduction

In recent decades, scientific advances in oncology and the greater availability of more effective systemic therapies have increased the survival of cancer patients and, with it, the prevalence of metastatic disease. 1 The spine is the most common site of skeletal metastases due to several factors, including its rich vascularization and abundance of bone marrow tissue. These lesions can cause pain, mechanical instability, and, in more severe cases, spinal cord (or cauda equina) compression, representing an interdisciplinary therapeutic challenge. The treatment of metastases is palliative, with different goals, such as pain relief, neurological recovery, quality of life improvement, tumor volume control, and systemic therapy availability.

Radiotherapy is a fundamental modality for managing spinal bone metastases. The development of advanced technologies has revolutionized the therapeutic scenario, allowing for more effective, safe, and individualized approaches. Although conventional external beam radiation therapy (cEBRT) remains the standard approach, recent years have witnessed the emergence of stereotactic body radiation therapy (SBRT) as an effective alternative, particularly for local control and radioresistant tumors. 1

The current article presents an updated review covering the history and innovations in treating spinal metastases, focusing on their benefits, indications, and clinical impact.

## Brief history of the evolution of spinal metastases treatment

Over the past 50 years, the palliative treatment of metastases has evolved towards achieving more lasting effects and lower morbidity. In the 1970s, surgical techniques still lacked refinement, and decompressions resulted in gross instabilities. In this context, cEBRT gained significance as a therapeutic strategy despite its low efficacy for solid tumors. There was the channeling of radiation in a fractionated manner due to the concern of sparing crucial organs, such as the bone marrow, digestive system, and kidneys, but with unsatisfactory outcomes in disease control. In 1978, Gilbert et al **.** 2 demonstrated the radiosensitivity and radioresistance of tumors based on their histology, with hormone-dependent and hematologic tumors being the most responsive to radiation.

Advances in instrumentation techniques in the 1990s resulted in surgery resuming its critical role in managing patients with spinal metastatic tumors, ushering in an era of major spinal resections and reconstructions. In the 2000s, the transition from materials such as stainless steel to titanium enabled better imaging assessments and, therefore, better planning and execution of complementary radiotherapy in cases in which surgery was imperative.

In 2005, Patchell et al. 3 published a prospective randomized trial comparing cEBRT with surgery followed by cEBRT in patients with high-grade spinal canal compression by radioresistant solid tumors. The arm of the study in which surgery followed radiotherapy showed much better outcomes. As such, this and several other studies led the Spine Oncology Study Group (SOSG) to recommend this combined approach as the most effective strategy for these cases. 3

Over the past 15 years, the development of SBRT has revolutionized the paradigm for managing secondary spinal lesions. By definition, it consists of administering high-dose, poorly fractionated radiation, ranging from 16 to 24 Gy in a single fraction or 24 to 40 Gy in 2 to 5 fractions. Radiation application uses conformational techniques, that is, with more precise demarcation of the target site aided by images, sparing adjacent healthy tissues. This strategy overcomes the tumor's histological radioresistance, leading to effective local biological control.

This advent has limited the role of large surgical resections of the past. Metastatic tumors that do not compress the spinal cord, are radioresistant, in oligometastatic disease, previously treated with en-bloc resections, now receive radiosurgery as the first line of treatment. While the other surgical indications have remained the same since Patchell's study, advances in radiotherapy have changed the surgical goal. While there was previously a concern with the volume for resection, separation surgery now stands out, creating a space of 2 to 3 mm between the neural structures and the tumor mass, allowing its safe and effective irradiation.

The major drawbacks of radiotherapy are toxicity and side effects. Although damage to healthy and noble tissues, neuritis, myelitis, and compressive vertebral fractures still occur, progressive technological refinement has reduced their frequency.

## Pathophysiology and Mechanism of Action

Bone metastases result from a highly complex biological process initiated by the hematogenous dissemination of tumor cells that, upon migrating from the primary site, invade adjacent tissues, enter the circulation, and preferentially lodge in the bone marrow. This bone microenvironment, rich in growth factors, such as transforming growth factor-beta (TGF-β) and platelet-derived growth factor (PDGF), promotes tumor cell proliferation. These cells secrete proteins, such as parathyroid hormone-related peptide (PTHrP), which activate osteoclasts through the receptor activator of nuclear factor-kappa B (RANK)/RANK ligand (RANKL) signaling, intensifying bone resorption. In turn, bone degradation releases more growth factors stored in the matrix, perpetuating a vicious cycle fueling tumor progression, intensifying tissue destruction, and amplifying nociceptive stimulation.

From a pathophysiological perspective, multiple mechanisms mediate pain in bone metastases. These mechanisms include peripheral nerve fiber compression, release of inflammatory mediators (such as interleukin-1 [IL-1], tumor necrosis factor-alpha [TNF-α], and prostaglandins), and increased intraosseous pressure due to tumor infiltration. Microfractures and bone deformities exacerbate these changes, amplifying nociceptive stimuli and promoting persistent pain with difficult clinical control. 4

Radiotherapy exerts multifaceted therapeutic effects in treating bone metastases, including tumor cell apoptosis induction, which reduces tumor burden and intraosseous pressure. In addition, there is a significant suppression of osteoclastic activity, stabilizing bone resorption and facilitating the tissue repair process. Radiotherapy also modulates the inflammatory microenvironment by reducing the presence of inflammatory cells and pro-inflammatory cytokines, which explains the rapid pain relief observed in many cases, often within 24 hours after the start of treatment.

Another significant effect is the stimulation of ossification in osteolytic lesions, which contributes to the partial restoration of bone structural integrity. In addition, there is evidence that radiotherapy can directly modulate the excitability of peripheral nerve fibers in the irradiated region, which attenuates neuropathic pain associated with bone metastases.

## Palliative conventional external beam radiation therapy

Traditionally, cEBRT uses doses such as 8 Gy as a single dose or fractionated regimens (20 Gy in 5 fractions or 30 Gy in 10 fractions). In 2011, the American Society for Therapeutic Radiology and Oncology (ASTRO) published an evidence-based guideline for the palliative treatment of bone metastases. Analysis of several prospective randomized clinical trials demonstrated equivalence in pain relief for these different dose regimens frequently used in patients with painful bone lesions without a prior history of radiotherapy, with acceptable and similar late toxicities. The retreatment rate due to pain recurrence was different and significantly higher in single-fraction treatment (8% versus 20%). 5 The updated guideline version, in addition to reinforcing the previous issues, recommends a one-month interval as the minimum safe period for reirradiation in the same region or lesion. 6

A later systematic review and meta-analysis reinforced the evidence regarding the equivalence of single-fraction radiotherapy (SFRT) compared to multiple-fraction radiotherapy (MFRT) regarding efficacy in controlling pain, pathological fracture, and spinal cord compression. Retreatment rates were better with MFRT over the follow-up period. 7 8 These studies show overall pain response rates of approximately 60% and complete response rates of 10 to 25% with palliative cEBRT, with a response durability of around 4 months. 9

## Palliative stereotactic body radiation therapy

Stereotactic body radiation therapy represents a milestone in spinal metastases treatment. This technique allows the administration of ablative doses with high precision, minimizing the exposure of critical structures close to the lesions, such as the spinal cord. In addition, dose escalation provides better pain control rates than cEBRT. A 2024 guideline from the European Society of Therapeutic Radiology and Oncology (ESTRO), after a systematic review of prospective and retrospective studies, reports postspinal SBRT overall and complete pain response rates of 83.2% and 43.5%, respectively. 10

A randomized phase II study compared the analgesic durability of SBRT (12–16 Gy in a single dose) to palliative conventional radiotherapy (30 Gy in 10 fractions) in patients with predominantly painful bone non-vertebral metastases. The study demonstrated that SBRT was non-inferior to the conventional treatment in the overall pain response rate and had similar acute toxicity and fracture rates. 11 These findings represent the first prospective randomized evidence suggesting that SBRT could become the standard of care for patients, with good performance status, longer life expectancy, and limited bone metastases.

To investigate the outcomes of ablative radiotherapy in symptomatic spinal metastases alone, Sahgal et al. 11 conducted a phase 2/3 study at centers in Canada and Australia. The authors randomized 229 patients to receive SBRT at a dose of 24 Gy in two fractions or cEBRT at 20 Gy in five fractions. Lesions required confirmation by magnetic resonance imaging, could not involve more than three contiguous vertebral segments, and cause no spinal cord or cauda equina compression; in addition, the spinal instability neoplastic score (SINS) should be lower than 12. Patients included had an Eastern Cooperative Oncology Group (ECOG) performance status of 0 to 2 and a pain score of 2 points or more on the Brief Pain Inventory. With a primary endpoint of complete pain response 3 months after radiotherapy, the study revealed a significant benefit of SBRT, as 35% of patients in this group showed a response compared with 14% in the cEBRT group. There was no significant difference in adverse events, including vertebral fracture risk, and the 6-month follow-up revealed a durable response. 12

More recently, the phase 3 NRG-Oncology/RTOG 0631 study compared the two radiotherapy techniques by randomizing 339 patients with up to three sites of vertebral metastases to receive treatment in a single fraction with either SBRT (16 or 18 Gy to the involved segment) or cEBRT (8 Gy, including additional vertebra superior and inferior to the affected one). The study included patients with minimal epidural lesions at least 3 mm away from the spinal cord. However, there was no significant difference in pain response between SBRT and cEBRT at 3 months (40.3% versus 57.9% respectively) and in adverse event rates. 9 The divergence between the results of these two studies remains the subject of discussion, reinforcing the biological difference between doses and the clinical impact of physical planning with less rigorous objectives.

In practical terms, SBRT is appropriate in the palliative setting for symptom control in selected patients with painful vertebral metastases, providing local control rates of over 85 to 90% in 1 to 2 years, even in radioresistant tumors. This effect is significant for patients who, despite having metastasis, have increasingly prolonged survival times as systemic treatments evolve. Therefore, the guidelines recommend SBRT mainly for patients who are not unstable (SINS > 12), with none or minimal epidural disease (Bilsky 0–1), with up to three contiguous vertebral segments in the treatment volume, and a prolonged life expectancy. In contrast, for patients with a low life expectancy and for whom the main objective is short-term pain control, conventional radiotherapy should be the first choice.

## Radiotherapy in patients with oligometastasis

Hellman and Weichselbaum introduced the concept of oligometastasis in the 1990s, proposing an intermediate state between localized and widely-disseminated disease. The concept is based on the idea that some patients have a limited number of metastases, which can be targeted by specific therapies, potentially altering the natural course of the disease. These therapies include local interventions, such as surgery and radiotherapy, and modern systemic therapies. Stereotactic body radiation therapy has emerged as one of the most significant tools in managing oligometastasis, offering high precision and efficacy in controlling lesions with minimal side effects.

Randomized phase 2 studies have shown the first evidence of the benefit of metastasis-directed treatment (MDT). Gomez et al. selected only patients with up to three metastases from primary non-small cell lung cancer to receive local consolidation therapy (surgery or SBRT) or maintenance therapy/observation. The study termination occurred early due to the favorable outcomes of local treatment, with a median progression-free survival (PFS) of 14.2 months (versus 4.4 months) and median overall survival (OS) of 41.2 months (versus 17 months). 13

In a broader analysis, including different primary neoplasms, the SABR-COMET study randomized patients to receive standard palliative treatment alone or combined with MDT and SBRT. Despite allowing up to five metastases, most (∼ 90%) subjects had up to three lesions, with approximately 33% being bone metastases. Despite the benefit in PFS and OS in patients who underwent SBRT for oligometastasis, the analysis of the results should be individualized due to the heterogeneity of the study population. 14

In clinical practice, SBRT uses different fractionations and doses, but evidence suggests that higher doses are associated with better local control. A randomized phase-3 study compared the outcomes of fractionated SBRT (27 Gy in 3 fractions) and single-dose SBRT (24 Gy in a single fraction). Of the 117 patients, 88% had bone oligometastasis, and 56% had spine metastasis. After a median follow-up time of 52 months, the local recurrence rate at 3 years was 5.8% with a single dose and 22% with 3 fractions of 9 Gy. In addition, patients treated with a single dose had a lower incidence of distant metastasis, with no significant increase in toxicity. Therefore, one must consider prescribing high equivalent doses to improve local control with careful analysis of the local toxicity risks. 15

## Spinal cord compression

Spinal cord compression causes a broad spectrum of symptoms. Its severity depends on presentation degree, ranging from asymptomatic to complete plegia. Thus, it is an oncological emergency, and its therapeutic management is critical for damage control and optimization for potential symptom reversion.

In this scenario, radiotherapy is well established and evaluated in classic studies in oncology. In 2005, Patchell assessed the clinical benefit of decompressive surgery and postoperative radiotherapy. Compared with patients treated with radiotherapy alone, the combined treatment resulted in better outcomes regarding functional neurological capacity. Among patients able to walk at baseline, 94% in the surgical group had preserved function after treatment compared with 74% in the cEBRT group. Of the 16 patients in each arm who could not walk, 62% recovered after combined treatment compared with 19% in the cEBRT group. In addition, treatment with surgery and radiotherapy led to a substantial reduction in the use of corticosteroids and opioid analgesics. 3

Regarding the radiotherapy dose used, the SCORE-2 study demonstrated the non-inferiority of a shorter treatment, with 5 fractions of 4 Gy, compared with a longer treatment, with 10 fractions of 3 Gy, for patients with metastatic epidural compression. Reducing the radiotherapy time by half did not change pain control, neither did it increase the progression rate or functional deterioration. 1 2 To further reduce treatment time, SCORAD III randomized patients to receive 20 Gy in 5 fractions and 8 Gy in a single fraction. Despite not meeting the non-inferiority criterion for the deambulatory status at 8 weeks (the primary study outcome), the evaluation at 1, 4, and 12 weeks revealed that the longer treatment was not inferior to the single-dose radiotherapy. 16 Therefore, depending on the individualized evaluation of each patient, a shorter treatment may be an effective alternative.

Some patients with spinal cord compression can receive SBRT to improve local control. However, epidural disease close to the spinal cord can compromise the likelihood of control, and, therefore, strategies such as separation surgery can make radiotherapy safer and more effective.

## Prophylactic radiotherapy in asymptomatic metastases

Metastatic bone lesions are often diagnosed in follow-up or staging tests and may not cause pain. Nonetheless, at some point in the patient's evolution, bone events resulting from these lesions may occur, such as pathological fracture, spinal cord compression, or the need for surgery or radiotherapy. A recently published randomized phase-2 study evaluated the benefits of prophylactic radiation in bone metastases. In this study, approximately 31% of patients had lesions in the spine region. Conventional external beam radiation therapy significantly reduced bone events, at a rate of 1.6% in 1 year, compared with 29% in patients not submitted to the initial irradiation. Therefore, a multidisciplinary team should evaluate and discuss the best therapeutic strategy for asymptomatic patients with high-risk lesions, such as in the junctional spine. 17

## Adverse effects

The most frequent adverse event after radiotherapy for painful bone metastases is acute pain worsening, with a higher risk after SBRT than cEBRT (43% and 33% respectively). 18 The increased risk also occurs in lesions with soft-tissue involvement and treatment volume > 8 cm ^3^ . 19

Vertebral compression fracture (VCF) is less frequent, with rates ranging from 6 to 39%. 20 Its risk factors include lytic lesions, location in the thoracic spine, preradiotherapy SINS > 8, preexisting VCF, and high single doses (20–24 Gy). This event can lead to different complications, such as pain, vertebral deformity, and spinal cord compression, sometimes requiring surgical correction. 21

The incidence of radiation myelopathy is low, at approximately 0.4% in patients who underwent the first irradiation in the spine. The dose reaching the spinal cord varies according to the degree of compression of the spinal canal, being higher when the compression is greater. More recent studies have established dose limits for organs at risk for treatment planning in an attempt to keep the risk below 5%. Reports on brachial and lumbosacral plexopathies are rare. 22

The emergence of treatments increasingly prolonging the survival of metastatic patients results in a higher possibility that some cases will need a reirradiation of bone lesions. In this scenario, SBRT toxicity may become more relevant. Likewise, radiotherapy association with new systemic treatments and the potential adverse events of this combination remain a subject of investigation. 23 Fig. shows an example of SBRT.

**Fig. 1:**
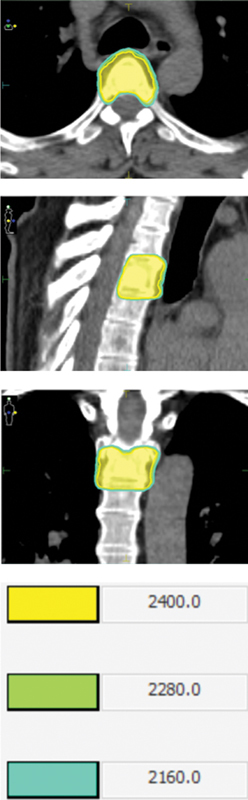
Example of treatment with stereotactic body radiation therapy (SBRT). The patient had primary prostate cancer. He presented biochemical recurrence, underwent radical treatment, and was followed up for 4 years. The subject presented disease progression in bone topography, with indication to SBRT for local control as an oligometastatic patient. Stereotactic body radiation therapy used 2 fractions of 1,200 cGy in the thoracic spine.

## Surgery

In the routine of spine surgeons working in the oncological setting, the neurologic, oncologic, mechanical, systemic (NOMS) algorithm, developed in 2004 and updated every 5 years with the most recent evidence, plays an important role. This flowchart considers the NOMS aspects to assist in the best decision-making for each case. 24

In 2005, Patchell et al. 3 established a paradigm for the performance of spine surgeons in the context of metastatic disease, establishing surgery as an intervention for radioresistant solid tumors causing high-grade spinal cord compression. The assessment of the compression degree occurs in axial T2-weighted magnetic resonance imaging. Its classification follows the Bilsky score, in which 0 indicates bone alone, 1 a, b, and c refer to compression of the dural sac with no spinal cord compression, 2 alludes to spinal cord or cauda equina compression but with visible cerebrospinal fluid, and 3 is the absolute canal compression. Scores of 2 and 3 reveal high-grade compression. 25

The surgical indications proposed by Patchell remain valid, but the objective of surgery for metastatic disease has been updated with the advancement of radiosurgery. In cases with high-grade compression by radioresistant tumors, irradiation can cause myelitis or permanent damage due to anatomical proximity, requiring an associated surgical approach, that is, the “separation surgery” model. This model limits tumor resection to neural tissue decompression, creating a space of 2 to 3 mm to provide a safe target for local radiotherapy. This approach also has a surgical benefit due to the smaller extent of tumor resection, potentially reducing surgical time and blood loss. 26

Surgery in the context of metastatic disease still considers the scenario of mechanical instability. In 2011, SOSG introduced SINS to aid in decision-making regarding treating secondary spinal lesions. In the last SINS assessment, scores can range from 0 to 6, denoting stability, 7 to 12, indicating indeterminate instability, and 13 to 18, revealing instability. Surgical stabilization is recommended for scores higher than 12; meanwhile, for scores lower than 7, initial treatment is non-surgical. Decision-making becomes challenging for most patients with scores from 7 to 12, that is, “potentially unstable” lesions. 27

## Final considerations

Recent advances in the diagnosis and systemic and local treatment of metastatic cancer have transformed the oncological practice, expanding the prospects for long-term overall survival for patients with metastases. This development has significant implications for palliative radiotherapy, which now encompasses not only immediate symptomatic relief but also prolonged disease control, focusing on minimizing the late effects of therapy and potentially contributing to cure in selected patients.

In this context, SBRT introduction emerges as a valuable tool in managing spinal metastases, allowing a targeted and effective approach. Stereotactic body radiation therapy offers benefits such as long-lasting pain control, prevention of local progression, and reduction of associated complications. In addition, it integrates effective strategies in oligometastatic patients. However, one should carefully consider escalated doses due to the potential risk of increased toxicity, the need for advanced technology and trained staff, and the time required for application compared with conventional radiotherapy.

As such, SBRT represents a promising advance in managing spinal metastases, especially in selected patients, in line with the demands of modern oncology, seeking to reconcile therapeutic efficacy, quality of life preservation, and individualized approaches.
